# The Effect of Finasteride and Dutasteride on the Growth of WPE1-NA22 Prostate Cancer Xenografts in Nude Mice

**DOI:** 10.1371/journal.pone.0029068

**Published:** 2012-01-05

**Authors:** Alexander B. Opoku-Acheampong, Michelle K. Nelsen, Dave Unis, Brian L. Lindshield

**Affiliations:** Department of Human Nutrition, Kansas State University, Manhattan, Kansas, United States of America; Queensland University of Technology, Australia

## Abstract

**Background:**

5α-reductase 1 (5αR1) and 5α-reductase 2 (5αR2) convert testosterone into the more potent androgen dihydrotestosterone. 5αR2 is the main isoenzyme in normal prostate tissue; however, most prostate tumors have increased 5αR1 and decreased 5αR2 expression. Previously, finasteride (5αR2 inhibitor) treatment begun 3 weeks post-tumor implantation had no effect on Dunning R3327-H rat prostate tumor growth. We believe the tumor compensated for finasteride treatment by increasing tumor 5αR1 expression or activity. We hypothesize that finasteride treatment would not significantly alter tumor growth even if begun before tumor implantation, whereas dutasteride (5αR1 and 5αR2 inhibitor) treatment would decrease tumor growth regardless of whether treatment was initiated before or after tumor implantation.

**Methodology/Principal Findings:**

Sixty 8-week-old male nude mice were randomized to Control, Pre- and Post-Finasteride, and Pre- and Post-Dutasteride (83.3 mg drug/kg diet) diet groups. Pre- and post-groups began their treatment diets 1–2 weeks prior to or 3 weeks after subcutaneous injection of 1×10^5^ WPE1-NA22 human prostate cancer cells, respectively. Tumors were allowed to grow for 22 weeks; tumor areas, body weights, and food intakes were measured weekly. At study's conclusion, prostate and seminal vesicle weights were significantly decreased in all treatment groups versus the control; dutasteride intake significantly decreased seminal vesicle weights compared to finasteride intake. No differences were measured in final tumor areas or tumor weights between groups, likely due to poor tumor growth. In follow-up studies, proliferation of WPE1-NA22 prostate cancer cells and parent line RWPE-1 prostate epithelial cells were unaltered by treatment with testosterone, dihydrotestosterone, or mibolerone, suggesting that these cell lines are not androgen-sensitive.

**Conclusion:**

The lack of response of WPE1-NA22 prostate cancer cells to androgen treatment may explain the inadequate tumor growth observed. Additional studies are needed to determine whether finasteride and dutasteride are effective in decreasing prostate cancer development/growth.

## Introduction

Prostate cancer is the most commonly diagnosed malignancy in men, estimated to account for nearly 30% of cancer cases in 2011 [1]. Prostate tumor growth is commonly stimulated by androgens. Testosterone, the main circulating androgen, is converted by the isoenzymes 5α-reductase 1 and 5α-reductase 2 into the more potent dihydrotestosterone, which binds with up to ten-fold higher affinity to the androgen receptor than testosterone [2], [3]. 5α-reductase 1 is the major isoenzyme in human liver and nongenital skin, whereas 5α-reductase 2 is the major isoenzyme in the prostate, epididymis, seminal vesicle, and genital skin [4].

Inhibiting androgen production and/or blocking its action are common approaches for combatting prostate cancer [5]. Most studies report increased 5α-reductase 1 and decreased 5α-reductase 2 mRNA expression or activity in prostate cancer [6]–[9]. Others have reported increased 5α-reductase 1 mRNA expression and no significant changes in 5α-reductase 2 mRNA expression in prostate cancer versus normal tissue [10], increased expression of both isoenzymes in prostate cancer [5], or loss of expression of both isoenzymes in metastatic prostate cancer [11]. Two 5α-reductase inhibitors, finasteride (5α-reductase 2 inhibitor) and dutasteride (5α-reductase 1 and 2 inhibitor), are commonly used to treat benign prostatic hyperplasia (BPH) [12]. These 5α-reductase inhibitors also could be used to prevent or treat prostate cancer by reducing dihydrotestosterone levels [13].

In support of this possibility, finasteride decreased prostate cancer prevalence by 24.8% in the Prostate Cancer Prevention Trial (PCPT) [14]. Similarly, in the Reduction by Dutasteride of Prostate Events (REDUCE) trial, dutasteride reduced prostate cancer incidence by 23% [15]; however, based on the results from these trials, the Food and Drug Administration (FDA) recently revised the safety information for both drugs to state that the drugs increase patients' risk for developing high-grade prostate cancer [16]. In animal models, dutasteride, but not finasteride, inhibited growth of Dunning R-3327H rat prostate tumors [17]. In nude mice bearing LNCaP human prostate cancer xenografts, both finasteride and dutasteride reduced tumor growth, although dutasteride was more effective at an equimolar dose [17]. In rats, finasteride significantly decreased androgen-sensitive tissue weights, but did not decrease Dunning R-3327H tumor growth [18].

In these animal studies, finasteride and dutasteride administration began after tumors were established; finasteride administration initiated before tumor implantation may be more efficacious. On the other hand, regardless of when finasteride treatment is initiated, prostate cancer cells may compensate for 5α-reductase 2 inhibition by increasing 5α-reductase 1 expression and/or activity; thus, the dual inhibitory effect of dutasteride may offer an advantage over finasteride. We examined the effect of finasteride and dutasteride diets begun 1 week before or 3 weeks after subcutaneous injection of WPE1-NA22 human prostate cancer cells into the rear flanks of male nude mice. We used WPE1-NA22 prostate cancer xenografts because these human cancer cells can be cultured *in vitro*, yet form noninvasive tumors with growth rates and pathology similar to the Dunning R-3327H tumor [19], [20].

## Materials and Methods

The Institutional Animal Care and Use Committee (IACUC) of Kansas State University approved all animal procedures (protocol 2794).

### Cell Lines

WPE1-NA22 prostate cancer and RWPE-1 prostate epithelial cells (ATCC, Manassas, VA) were cultured in serum-free keratinocyte media containing bovine pituitary extract and epidermal growth factor (GIBCO Invitrogen, Carlsband, CA). For the animal study, WPE1-NA22 cells were cultured in 75 cm^2^ flasks (Fisher Scientific, Pittsburg, PA), removed with trypsin (Sigma-Aldrich, St. Louis, MO), and centrifuged for 7 minutes at 130×g at 37°C. Supernatant was removed and cells were reconstituted in Matrigel™ (BD Biosciences, Franklin Lakes, NJ) at a concentration of 5,000 cells/µL. Twenty microliters of Matrigel™ containing ∼1×10^5^ WPE1-NA22 cancer cells was injected into each rear flank of nude mice using a Hamilton syringe holder (Hamilton, Reno, NV) fitted with a 1 mL syringe and a 25 gauge 5/8-in. needle (both from BD Biosciences).

### Animals, Study Diets and Design

**Figure 1:**
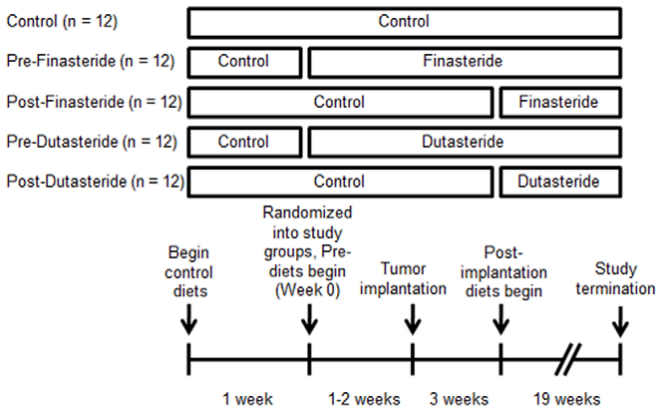
Animal study design: After receipt, mice were acclimated for 1 week then randomized into Control, Pre-Finasteride, Post-Finasteride, Pre-Dutasteride, and Post-Dutasteride groups (n = 12). Pre- and post-groups began their treatment diets 1–2 weeks prior or 3 weeks after WPE1-NA22 cell injection, respectively. The study was terminated 22 weeks post-tumor implantation.

Two cohorts of 30 (60 total) 8-week-old male nude mice (Charles Rivers, Wilmington, MA) were individually housed in sterile conditions. Mice were monitored daily, weighed weekly, and provided diets and water *ad libitum*. AIN93-G treatment diets (Research Diets, New Brunswick, NJ) contained dutasteride (provided by GlaxoSmithKline Pharmaceuticals, Research Triangle Park, NC) and finasteride (Kemprotec, Middlesbrough, UK) at 83.3 mg/kg of diet, designed to provide ∼10 mg drug/kg body weight, which was the middle dutasteride dose used by Xu and colleagues [17]. After receipt, mice were acclimated for 1 week before being randomized to Control, Pre-Finasteride, Post-Finasteride, Pre-Dutasteride, and Post-Dutasteride groups (n = 10–12, Figure 1). Five mice did not complete the study for health reasons unrelated to tumor growth. Pre- and post-groups began their treatment diets 1–2 weeks prior or 3 weeks after WPE1-NA22 cell injection, respectively. The three weeks after injection timepoint was chosen because Canene-Adams and colleagues initiated finasteride treatment at the same point [18]. The study was terminated 22 weeks post-tumor implantation. Mice were euthanized by CO_2_ inhalation and blood was immediately drawn via cardiac puncture and centrifuged for 1 minute at 2000×g to obtain serum. Tissues were dissected, flash frozen in liquid nitrogen, and stored in a freezer at −80°C.

Tumor area was calculated using the formula for area of an ellipse: area  =  π*(length/2)*(width/2). The average tumor area in a group was calculated by summing the individual tumor areas for the group, then dividing by the total number of tumor sites in the group. Zeros were recorded for tumor sites without measureable tumors.

### *In Vitro* Androgen Treatment and Cell Viability

WPE1-NA22 cells (passage number ≤7) and RWPE-1 cells (passage number ≤6) were plated at 10,000 cells per well in 96-well plates (Fisher Scientific, Pittsburg, PA). Twenty-four hours after plating, both cell lines were treated with testosterone (0.1 nM–30 nM), dihydrotestosterone (0.03 nM–100 nM, both from Sigma Aldrich, St. Louis, MO), and synthetic androgen mibolerone (0.01 nM–20 nM, PerkinElmer, Waltham, MA) in 0.1% ethanol. Media and androgen treatments were prepared daily and changed every 24 hours during the 5-day treatment period. Cell viability was quantified using the CellTiter 96 AQueous One Solution Assay (Promega Corporation, Madison, WI) with a Bio-Tek uQuant Plate reader (BioTek, Winooski, VT). Results presented are from 4 replicates of the experiments.

### Statistical Analysis

Data was analyzed using SAS 9.2 (SAS Institute Inc., Cary, North Carolina), with p<0.05 considered statistically significant. ANCOVA with cohort as the covariate was used to initially analyze the animal study results. The covariate was removed because it did not account for a significant amount of variance in all analyses, and ANOVA with Tukey's test was used on pooled data from the two cohorts. Natural logs were used to transform data when data did not meet model assumptions. Kruskal Wallis non-parametric one-way ANOVA was used for tumor incidence. Androgen treatment cell viability data were analyzed using ANOVA with Dunnett's test.

## Results

**Figure 2:**
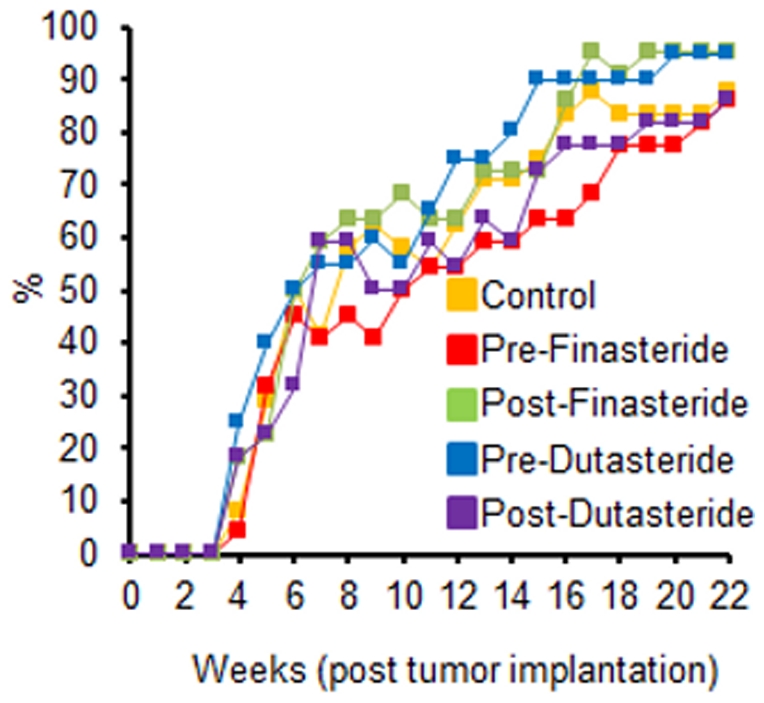
Tumor incidence (n  = 20–24) in tumor sites. Zeros were recorded for tumor sites without a tumor; no significant differences between groups.

**Figure 3:**
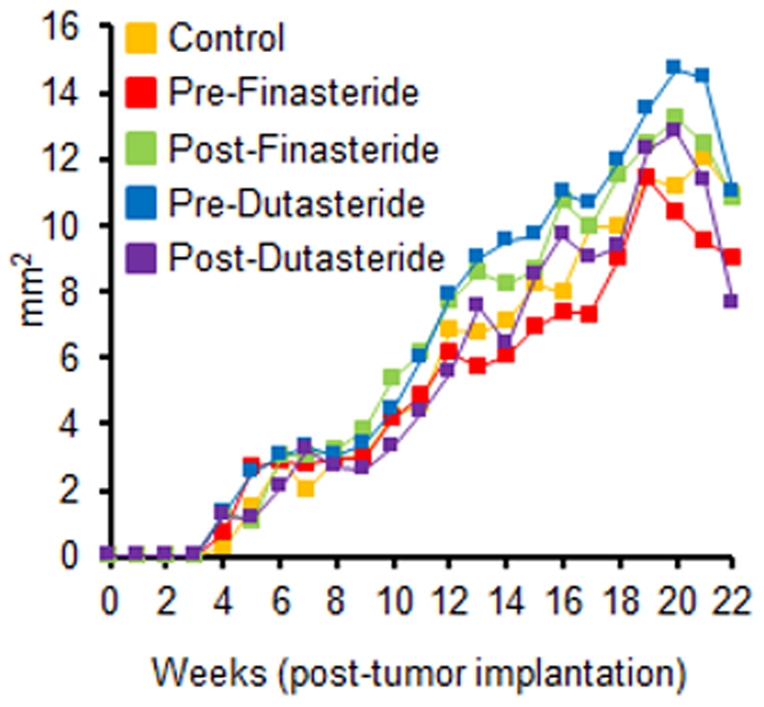
Tumor area (n = 20–24) of tumor sites. Zeros were recorded for tumor sites without tumors; no significant differences between groups.

Final body weights of the Pre-Finasteride group were significantly higher than the control (Table 1, p<0.05) despite no differences in daily food intake or food efficiency (data not shown) among the groups. Tumor incidence was high, 86.4% to 95.5%, with no difference between groups (Figure 2). No difference was found in tumor weights and tumor areas between groups (Table 1 and Figure 3), likely a result of poor tumor growth. The largest average tumor diameter in any group was 4.33 mm. Despite not altering tumor growth, both finasteride and dutasteride significantly decreased prostate and seminal vesicle weights as a percentage of body weight (p<0.05). In addition, there was a significant decrease in seminal vesicle weights in dutasteride groups versus finasteride groups (Table 1).

**Table 1:** Final body weights, tumor incidence, tumor weights, seminal vesicle weights as a percentage of body weight, and prostate weights as percentage of body weight^1^.

Groups	Final body weights (g, n = 10–12)	Final tumor incidence (%, n = 20–24)	Tumor weights (mg, n = 20–24)	Seminal vesicle weights (% body weight, n = 10–12)	Prostate weights (% body weight, n = 10–12)
Control	30.6 ± 0.6^a^	87.5	35 ± 7	0.92 ± 0.05^a^	0.42 ± 0.05^a^
Pre-Finasteride	33.2 ± 0.7^b^	86.4	25 ± 3	0.34 ± 0.02^b^	0.23 ± 0.02^b^
Post-Finasteride	30.9 ± 0.8^a^	95.5	30 ± 4	0.38 ± 0.03^b^	0.27± 0.02^b^
Pre-Dutasteride	29.4 ± 0.9^a^	95.0	36 ± 8	0.21 ± 0.01^c^	0.26 ± 0.02^b^
Post-Dutasteride	30.2 ± 0.6^a^	86.4	22 ± 2	0.23 ± 0.02^c^	0.23 ± 0.03^b^

1 Data are means ± SEM; values with different letters are statistically different.

**Figure 4:**
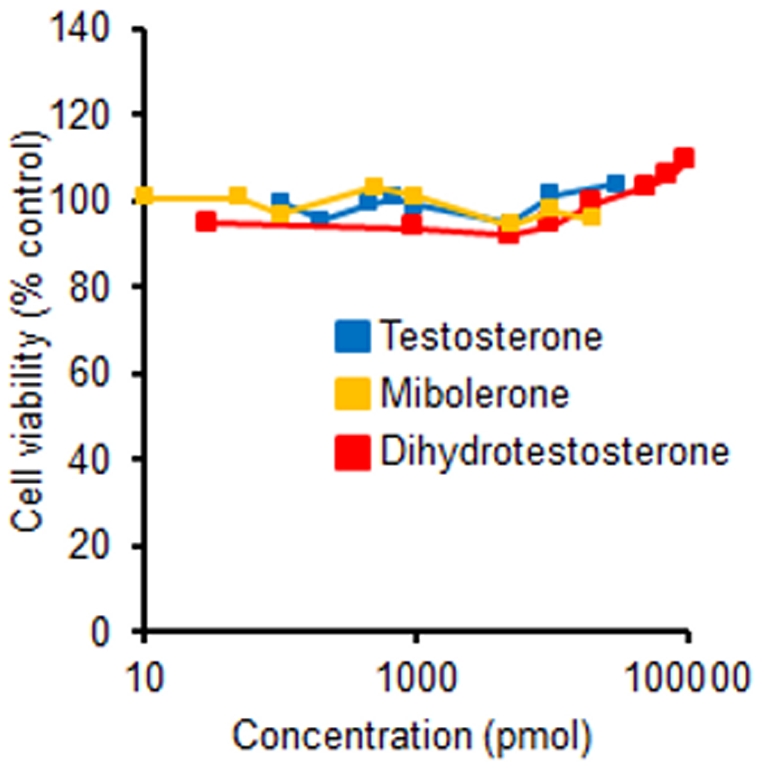
RPWE-1 (10,000 cells/well) cell viability was not altered by daily treatment of testosterone (0.1 nM–30 nM), dihydrotestosterone (0.03 nM–100 nM), or mibolerone (0.01 nM–20 nM) after a 5-day treatment period; no significant treatment effects.

**Figure 5:**
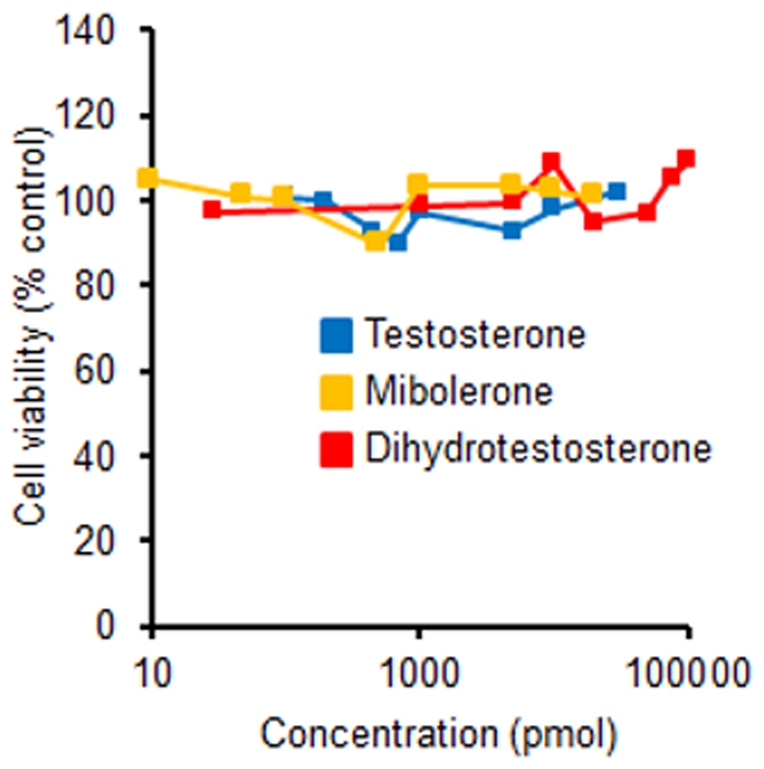
WPE1-NA22 (10,000 cells/well) cell viability was not altered by daily treatment of testosterone (0.1 nM–30 nM), dihydrotestosterone (0.03 nM–100 nM), or mibolerone (0.01 nM–20 nM) after a 5-day treatment period; no significant treatment effects.

These reductions in androgen-sensitive tissues suggest that finasteride and dutasteride were exerting their anti-androgenic action. One explanation for the poor growth is that WPE1-NA22 cells are not androgen-sensitive like their parent RWPE-1 human prostate epithelial cells [21]. Thus, WPE1-NA22 and RWPE-1 cells were treated with varying concentrations of testosterone, dihydrotestosterone, and the synthetic androgen mibolerone. We found no difference in cell numbers in either cell line when treated with varying concentrations of androgens (Figures 4 and 5).

## Discussion

In this study, we examined the effects of two 5α-reductase inhibitors, finasteride and dutasteride, pre- and post-tumor injection on the growth of WPE1-NA22 xenografts in nude mice. Tumor incidence was high for all groups ranging from 86.4% to 95.5%, similar to the ∼92–99% reported in previous Dunning R-3327H rat prostate cancer studies [18], [22]. The poor growth of WPE1-NA22 xenografts in nude mice was a surprise given that the xenograft tumor volume was previously reported to be 0.2 cm^3^ 7 weeks after implantation [20]. Back-calculating this is a tumor diameter of ∼7.26 mm, which is much larger than we observed at any time during our study.

One methodological difference between studies that likely contributed to the differences in tumor growth is the difference in the number of WPE1-NA22 cancer cells injected into the flanks of mice. Webber and colleagues subcutaneously injected 5×10^5^ WPE1-NA22 cells, five times more cells than we injected in this study [20]. Fewer cells were injected because of concerns that the tumor growth would be too rapid, given the size reported at 7 weeks compared with Dunning R-3327H tumors that are not palpable until 9–10 weeks post-tumor implantation [18], [22]. Two other possible explanations for the poor growth of WPE1-NA22 xenografts are that the nude mice were generating an immune response against the cancer cells or that the cells were not androgen-sensitive, so androgens did not stimulate their growth.

We set out to investigate the latter possibility by treating WPE1-NA22 cells and their parent cell line, RWPE-1 human prostate epithelial cells with a variety of concentrations of three androgens. Previously, growth of RWPE-1 cells increased in a dose-dependent manner when treated with mibolerone at doses of 0.01–10 nM [21]. Androgen concentrations used in our *in vitro* studies were based on several studies that examined the proliferative response to the three androgens [21], [23]–[25]. Physiological intraprostatic levels of both testosterone (∼0.2 nM–0.7 nM) [26] and dihydrotestosterone (5 nM–18 nM) [17], [27], [28] in humans fall within the range of concentrations used. For mibolerone, we used the concentrations by Bello and colleagues [21], but we also doubled their top concentration for our highest concentration.

Interestingly, we found no difference in cell numbers in either cell line in response to various concentrations of respective androgen treatments. We tried to repeat the methodology of Bello and colleagues; the only difference being that we used the MTS assay whereas they used a methylene blue assay for quantitating cell viability [21].

The lack of androgen sensitivity of both cells may explain the observed poor tumor growth. Further supporting our findings are that nuclear androgen receptor, nuclear 5α-reductase 2, and cytosolic 5α-reductase 1 protein levels are undetectable in RWPE-1 cells [29]. Cell lines derived from this parent line likely have similar levels of these key androgen metabolism/action proteins. Furthermore, both cell lines are grown in media without fetal bovine serum and exogenous androgens, meaning it is androgen-free. Taken together, these results should be considered before using or interpreting results from RWPE-1 and its carcinogenic derived cell lines.

Another surprising result was the significantly higher body weight in the Pre-Finasteride group without an alteration in food intake or food efficiency. Based on the trend in growth in the Pre-Finasteride group, we believe the group was heavier at randomization even though the differences in body weights were not significant at the time. The Pre-Finasteride group was already significantly heavier than the Pre-Dutasteride group 1 week after randomization despite negligible differences in food intake (the Pre-Dutasteride group consumed numerically more during that week), which supports our belief. Finasteride has been found to slightly increase weight gain in men with prostate cancer [30], but long-term intake did not increase the body weights of rats [31]. Diets in all treatment groups were well tolerated with no noted adverse effects.

The magnitude of decrease of prostate and seminal vesicles weights in response to dutasteride and finasteride were similar to those reported previously [18], [32], [33]. We also found a significant decrease in seminal vesicle weights in dutasteride groups versus finasteride groups. To the best of our knowledge, we are the first to report that dutasteride results in a greater magnitude of decrease in seminal vesicles weights than finasteride. Mice in our study consumed approximately 13 mg/kg/day of finasteride or dutasteride, which is greater than the 5 mg/kg/day of finasteride used by Canene and colleagues [18] but similar to the middle dutasteride dose used by Xu and colleagues [17].

In summary, although we did not see an effect of both inhibitors on the growth of WPE1-NA22 xenograft *in vivo*, results from the prostate and seminal vesicles indicate that the inhibitors were effective in inhibiting their respective 5α-reductase enzyme(s). Further research in different models will be required to answer our research question; however, our results question the tumorigenicity of WPE1-NA22 cells in nude mice and the androgen-sensitivity of WPE1-NA22 and RWPE-1 cells.
